# Influence of PLA Flowability and Talc Content on the Performance of Rigid TPS/PBS/PLA/Talc Blends

**DOI:** 10.3390/polym18121544

**Published:** 2026-06-21

**Authors:** Cristina Martín-Poyo, Josep P. Cerisuelo, Jose D. Badia-Valiente

**Affiliations:** 1 BZERO, Camino de Vera s/n, 46022 Burjassot, Valencia, Spain; 2 Research Group in Materials Technology and Sustainability (MATS), Department of Chemical Engineering, School of Engineering, Universitat de València, Av. Universitat s/n, 46100 Burjassot, Valencia, Spain

**Keywords:** thermoplastic starch, poly(butylene succinate), polylactide, talc, polymer blends, injection moulding, rigid packaging

## Abstract

This study investigates the influence of PLA flowability and talc content on the performance of compostable thermoplastic starch/poly(butylene succinate) (TPS/PBS)-based systems for rigid applications. Different PLA grades with varying melt flow index (PLA23, PLA8 and PLA70) and talc contents (0, 5 and 10 wt%) were incorporated. Twelve formulations were compounded by twin-screw extrusion and processed by injection moulding. FTIR confirmed the coexistence of TPS, PBS and PLA phases without evidence of chemical interactions. Morphological analysis showed that PLA flowability plays a key role in phase distribution, with higher-flow PLA promoting improved dispersion and interfacial adhesion, while talc addition (5 and 10 wt%) increased structural heterogeneity; at higher loadings, particularly, DSC analysis revealed that talc acted as a nucleating agent for the PBS phase, increasing crystallisation temperatures from approximately 73 °C to 81 °C depending on formulation. Mechanical results showed that Young’s modulus increased from approximately 1.4 GPa to 2.7 GPa with decreasing PLA flowability and increasing talc content. Formulations containing low-flow PLA reached tensile strengths close to 32 MPa, although elongation at break decreased to values near 2%. In contrast, high-flow PLA formulations exhibited a more balanced mechanical response, with elongation values up to approximately 8%, associated with improved phase dispersion. Hybrid PLA systems showed intermediate behaviour, reaching elongations up to 22% while maintaining modulus values around 1.8 GPa. Talc provided additional reinforcement but reduced deformation capacity. HDT values remained relatively constant, indicating limited improvement in thermomechanical resistance despite increased stiffness. These results demonstrate that the combined control of PLA molecular characteristics and talc content enables tuning of the mechanical and thermomechanical performance of TPS/PBS/PLA/talc systems for rigid packaging applications.

## 1. Introduction

The environmental persistence of conventional fossil-based plastics has become one of the most pressing challenges associated with modern material consumption. The extensive use of non-biodegradable polymers such as polyethylene, polypropylene, and poly(ethylene terephthalate) has led to the severe accumulation of plastic waste in terrestrial and marine environments, while current recycling strategies remain insufficient to fully mitigate this impact [1]. As a consequence, biodegradable and compostable polymers, ensuring long-term properties [2], have gained increasing attention as promising alternatives, particularly for high-volume applications such as packaging and disposable products [3]. However, the widespread adoption of these materials remains limited due to their higher cost, narrower processing windows, and, in many cases, inferior mechanical and thermomechanical performance compared to conventional plastics [1,3].

Among biodegradable materials, starch-based polymers represent one of the most attractive options due to their abundance, renewability, and low cost. Through the incorporation of low-molecular-weight plasticizers such as glycerol, native starch can be transformed into thermoplastic starch (TPS), enabling processing by conventional melt-based technologies including extrusion and injection moulding [4]. Despite these advantages, TPS exhibits intrinsic limitations related to its high hydrophilicity and consequent moisture sensitivity, poor dimensional stability, and limited mechanical strength, which significantly restrict its direct use in applications requiring long-term stability or structural integrity [5].

To overcome these limitations, TPS has been extensively blended with biodegradable polyesters [6], aiming to combine the economic and environmental advantages of starch with the improved mechanical strength, thermal stability, and processability of polyesters [7,8,9,10,11]. In this context, several strategies have been proposed to improve TPS compatibility with more hydrophobic polymer matrices, including the chemical modification of starch and reactive blending approaches [12,13]. In particular, citric acid has been shown to promote esterification reactions with starch hydroxyl groups, reducing its hydrophilicity and improving interfacial cohesion without compromising biodegradability [14].

Among biodegradable polyesters, polylactic acid (PLA) has been one of the most widely investigated due to its high stiffness, transparency, and industrial relevance [15]. PLA is currently the most commercially established bio-based and biodegradable polymer, making it an attractive candidate for partially replacing conventional plastics in rigid and semi-rigid applications. Nevertheless, PLA exhibits an intrinsically brittle behaviour and limited heat resistance, particularly in injection-moulded parts, where its low crystallisation rate often results in poor thermomechanical performance [16,17,18]. PLA is currently being increasingly adopted in packaging, food service products, disposable items and semi-structural applications due to its bio-based origin, industrial compostability and compatibility with conventional processing technologies such as extrusion and injection moulding. In particular, rigid and semi-rigid packaging sectors represent one of the most promising industrial fields for PLA-based biodegradable systems, where tailored stiffness, dimensional stability and processability are required.

Blending PLA with TPS has been proposed as an effective strategy to reduce material cost and tailor mechanical behaviour. However, TPS/PLA blends frequently suffer from limited compatibility caused by the polarity mismatch between the hydrophilic TPS phase and the more hydrophobic PLA matrix. Recent studies on TPS/PLA systems have confirmed that interfacial incompatibility remains one of the main limitations governing the mechanical performance of these biodegradable blends, particularly under tensile loading, where poor phase adhesion generally results in limited elongation at break and premature failure. This incompatibility typically leads to phase-separated morphologies with weak interfacial adhesion, which negatively affects strength and elongation at break [15]. Several studies have shown that, despite formulation improvements, TPS/PLA systems processed by injection moulding still present challenges related to interfacial adhesion and mechanical balance [18,19].

Beyond PLA, increasing attention has been devoted to alternative biodegradable polyesters such as poly(butylene succinate) (PBS) and its bio-based counterpart (BioPBS). PBS is an aliphatic polyester characterised by higher ductility and improved thermal resistance compared to PLA, with mechanical properties often described as analogous to those of low-density polyethylene. These features make PBS particularly attractive for applications requiring toughness and dimensional stability, especially when processed by injection moulding [20]. Previous works have demonstrated that PBS-based systems can provide a more favourable balance between stiffness and toughness than PLA-based counterparts, although challenges related to phase morphology and dispersion persist when PBS is combined with highly polar components such as TPS [21]. The use of BioPBS further enhances the sustainability profile of these systems by incorporating bio-based succinic acid while maintaining good processability and mechanical performance [21].

In injection moulding applications, material design becomes particularly complex due to the strong coupling between formulation, rheological behaviour, and processing conditions. Parameters such as melt viscosity, mould filling capability, shrinkage, crystallisation kinetics, and final thermomechanical performance are all strongly influenced by blend composition and by the molecular characteristics of each component [1]. In this context, the grade of PLA, especially its melt flow index (MFI) and molar mass, plays a critical role in determining processing stability and final part performance. High-MFI PLA grades can improve flowability and mould filling but may compromise mechanical strength, while lower-MFI grades generally exhibit higher molar mass and improved mechanical properties at the expense of processability [14]. Despite its relevance, the systematic influence of PLA grade in multicomponent TPS-based systems designed for injection moulding remains largely unexplored.

In addition to blend formulation, the incorporation of mineral fillers is a common strategy for tailoring the mechanical and thermomechanical performance of injection-moulded biodegradable polymers. Talc is one of the most widely used mineral fillers due to its plate-like morphology, availability, and ability to act both as a reinforcing agent and as a nucleating agent. In PLA- and PBS-based systems, talc has been shown to accelerate crystallisation, increase stiffness, and significantly improve heat deflection temperature, which is a critical parameter for rigid applications [1]. However, excessive talc loading or inadequate dispersion can lead to embrittlement and stress concentration, negatively affecting toughness and elongation at break [22]. Therefore, the effect of talc must be carefully optimised within each specific polymer system and processing route.

Although extensive literature exists on TPS/PLA and TPS/PBS systems processed by extrusion or film blowing [8,15], studies specifically focused on TPS/BioPBS/PLA formulations designed for injection moulding remain limited. This is mainly due to the processing challenges associated with TPS-based systems, including their thermal sensitivity, narrow processing window, moisture sensitivity and limited compatibility with hydrophobic biodegradable polyesters. In addition, the high cooling rates involved in injection moulding may promote brittleness, internal stresses and morphological heterogeneity, burdening the production of dimensionally stable rigid pieces. In particular, there is a lack of systematic investigations simultaneously addressing the influence of PLA grade, talc content, and injection moulding processing, while avoiding the use of reactive compatibilizers that may increase formulation complexity or cost. This gap limits the establishment of clear design guidelines for the development of compostable materials suitable for rigid and semi-rigid injection-moulded applications. Therefore, the novelty of the present work lies not in the individual materials themselves, but in the systematic understanding of how PLA molecular characteristics, flowability and talc incorporation jointly affect the morphology, crystallisation behaviour and mechanical response of multicomponent TPS/BioPBS systems processed by injection moulding.

In this context, the aim of this study was to evaluate how PLA flowability and talc content affect the morphology, thermal behaviour, mechanical performance and thermomechanical stability of injection-moulded TPS/BioPBS/PLA systems intended for rigid and semi-rigid biodegradable packaging.

## 2. Materials and Methods

### 2.1. Materials

Native starch from potato was provided by Avebe S.L (Veendam, The Netherlands), and glycerol (99.5% purity) was purchased from Quimidroga (Valencia, Spain). Talc, LUZENAC HAR^®^ T84 (Mg_3_Si_4_O_10_(OH)_2_) was provided by Imerys Performance Minerals EMEA (Paris, France). BioPBS™ (FZ71PM grade) was supplied by PTT MCC Biochem Company Limited (Bangkok, Thailand). Poly(lactic acid) (PLA) grades were supplied by TotalEnergies Corbion (Gorinchen, The Netherlands). Three PLA grades with different melt flow indices (MFI) were used in order to evaluate the influence of polymer flowability, namely Luminy^®^ L175 (8 g/10 min), Luminy^®^ L130 (23 g/10 min) and Luminy^®^ L105 (70 g/10 min), and they were measured according to ISO 1133-1:2022 [23] (210 °C/2.16 kg). All PLA grades presented a density of 1.24 g/cm^3^ and melting temperatures close to 175 °C.

### 2.2. Melt Compounding and Injection Moulding

The different formulations, whose compositions are detailed in Table 1, were prepared by melt compounding using a co-rotating twin-screw extruder (Leistritz ZSE 27 MAXX—60D, L/D = 60, Nuremberg, Germany). The extrusion process was conducted at a constant production rate of 10 kg·h^−1^, a screw speed of 350 rpm, and under vacuum conditions (−0.7 bar) to ensure effective devolatilization. A progressive temperature profile was applied along the extruder, ranging from 45 °C in the feeding section to 185 °C in the final mixing and extrusion zones. Native starch was introduced in the main feeding zone (Barrel 1) as the primary component, while glycerol was added in Barrel 3 as a plasticizer to facilitate starch gelatinization and to form the thermoplastic starch (TPS) matrix. The initial processing section was configured with high-shear and mixing elements to promote starch destructurization and plasticization.

In the subsequent polymer blending stage, the polymeric components and mineral filler were introduced via a side feeder located at Barrel 9. Depending on the formulation, one or two PLA grades (selected from three different PLA grades used in this study. Preliminary trials revealed that blends combining PLA8 and PLA70 exhibited poor interfacial adhesion and severe phase separation, resulting in insufficient material integrity and preventing reliable sample collection and further characterisation; therefore, these formulations were excluded from the study) were incorporated simultaneously with BioPBS FZ71 and talc, allowing their direct incorporation into the molten TPS matrix. The downstream barrel zones contained distributive and dispersive mixing elements to ensure effective dispersion and homogeneous blending of all constituents. A vacuum degassing system was applied in Barrel 13 to remove residual moisture and volatile compounds, thereby improving melt stability and preventing processing defects. The compounded material was extruded through a strand die with a diameter of 5 mm, water-cooled, and pelletized into granules with a size range of 3–5 mm.

**Figure 1:**
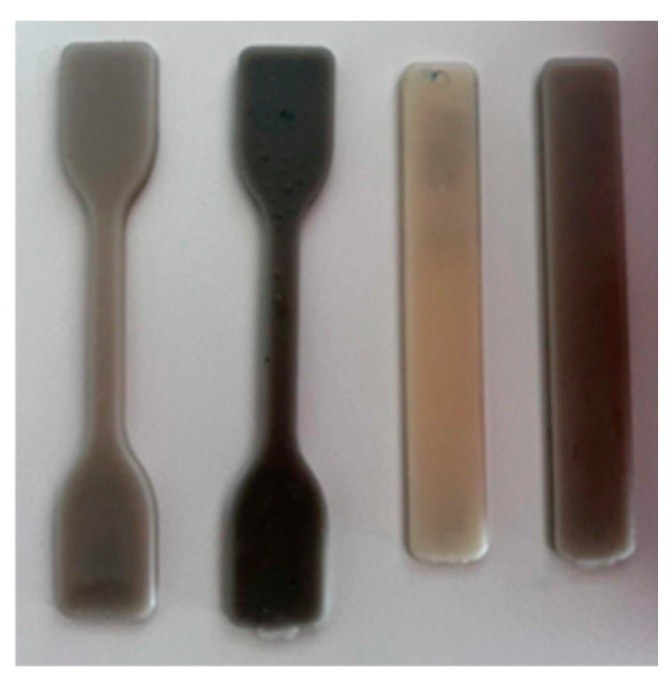
Geometry and dimensions of the injection-moulded specimens.

The resulting pellets were subsequently processed by injection moulding using an injection moulding machine (Battenfeld HM 45/210, Wittmann Battenfeld, Kottingbrunn, Austria) equipped with a 22:1 L/D screw and a maximum clamping force of 45 t to produce standard test specimens. Additional rectangular bars were also produced for subsequent HDT analysis. Injection moulding was performed under processing conditions adapted to each formulation in order to ensure stable melt flow, complete cavity filling and defect-free specimens. The barrel temperature profile ranged from 160 to 185 °C. The mould temperature, injection pressure and cooling time were adjusted depending on the formulation, within typical processing ranges of 25–40 °C for mould temperature, 50–80 MPa for injection pressure and 20–40 s for cooling time. Representative injection moulding conditions included injection pressures close to 900 bar, holding pressures up to 1250 bar and cooling times of approximately 45 s. Prior to mechanical and thermal characterisation, all injection-moulded samples were conditioned for 24 ± 1 h at 23 °C and 50% relative humidity to ensure consistency and comparability of the experimental results. Representative injection-moulded specimens used for tensile and HDT testing are shown in Figure 1.

### 2.3. Analytical Characterisation

#### 2.3.1. Fourier-Transform Infrared Spectroscopy (FTIR)

Fourier-transform infrared (FTIR) spectroscopy analyses were carried out using an Agilent Technologies Cary 630 FTIR spectrometer equipped with an attenuated total reflectance (ATR) accessory. For each formulation, spectra were collected from at least three independent specimens to ensure reproducibility. The measurements were performed over the wavenumber range of 4000–400 cm^−1^, using a spectral resolution of 4 cm^−1^ and averaging 32 scans per spectrum.

The FTIR analysis focused on identifying the characteristic absorption bands of the constituent materials and assessing potential interactions induced by variations in PLA grade and filler content. Particular attention was paid to the carbonyl stretching band around 1710 cm^−1^, associated with ester groups from PLA and BioPBS; the broad hydroxyl stretching band near 3400 cm^−1^, mainly related to starch and plasticizer components; the band at approximately 1580 cm^−1^, attributed to carboxylate groups; and the region around 1047 cm^−1^, characteristic of glycosidic linkages in starch. Changes in band intensity and shape were qualitatively analysed to infer structural modifications and interfacial interactions within the blends.

#### 2.3.2. Differential Scanning Calorimetry (DSC)

Thermal behaviour was evaluated by differential scanning calorimetry (DSC) using a Setaram Setline + DSC. The analyses were conducted under a nitrogen atmosphere to prevent oxidative degradation. Samples were sealed in aluminium pans and subjected to a heating–cooling–heating cycle from −50 °C to 220 °C at a constant rate of 10 °C·min^−1^.

At least two specimens per formulation were analysed to ensure the reliability of the detected thermal transitions. The second heating scan was used for comparative analysis in order to eliminate the influence of previous thermal history. Glass transition temperatures, melting behaviour and crystallisation phenomena were examined to evaluate the influence of the PLA grade and talc content on the thermal response of the injected materials, particularly on the crystallisation behaviour of the BioPBS phase.

#### 2.3.3. Scanning Electron Microscopy (SEM)

The fracture surface morphology of the injection-moulded specimens was examined by scanning electron microscopy (SEM) using a Philips XL 30 ESEM microscope equipped with an EDAX PV 9760 system. Prior to observation, the specimens were cryo-fractured in liquid nitrogen to obtain clean fracture surfaces and the samples were mounted on aluminium stubs using double-sided carbon adhesive tape and sputter-coated with a thin gold/palladium layer to ensure electrical conductivity.

SEM observations were carried out at an accelerating voltage of 10 kV and a magnification of 1000×. The analysis focused on evaluating phase dispersion, interfacial adhesion and fracture mechanisms, as well as identifying morphological differences associated with the PLA grade and formulation composition.

#### 2.3.4. Tensile Testing

Tensile properties were determined following the ASTM D638-22 [24] standard using a Testometric M350-20CT universal testing machine. Injection-moulded dog-bone specimens were tested at a crosshead speed of 50 mm·min^−1^ under controlled environmental conditions (23 °C and 50% relative humidity). Prior to testing, all specimens were conditioned under the same atmosphere to minimise variability.

From the nominal tensile stress (σ)—strain (ɛ) curves, Young’s modulus (GPa), tensile strength at break (MPa) and elongation at break (%) were determined.

#### 2.3.5. Flexural Testing

Flexural properties were evaluated by three-point bending tests in accordance with ASTM D790-23 [25] using the same universal testing machine. Injection-moulded rectangular specimens were tested under controlled environmental conditions (23 ± 2 °C) with a constant crosshead speed in the range of 1–2 mm·min^−1^, selected according to specimen dimensions and standard recommendations. At least five specimens per formulation were tested to ensure reproducibility of the results.

Flexural modulus, maximum flexural stress and deformation at maximum load were obtained from the resulting load–deflection curves. The flexural results were used to complement the tensile data and to assess the stiffness and load-bearing performance of the materials under bending conditions relevant to structural and semi-structural applications.

#### 2.3.6. Heat Deflection Temperature

The heat deflection temperature (HDT) was determined to assess the thermomechanical behaviour of the materials under load at elevated temperatures. The tests were carried out according to ISO 75-1:2020 [26] using an HDT/Vicat testing apparatus.

Injection-moulded rectangular specimens were subjected to a constant flexural stress of 1.8 MPa while being heated at a constant rate of 2 °C·min^−1^ under controlled conditions. Prior to testing, the specimens were conditioned at 23 °C and 50% relative humidity to ensure consistent testing conditions.

The HDT was defined as the temperature at which the specimen reached a deflection of 0.25 mm under the applied load.

At least five specimens per formulation were tested, and the average values were reported.

## 3. Results

### 3.1. Morphology

**Figure 2:**
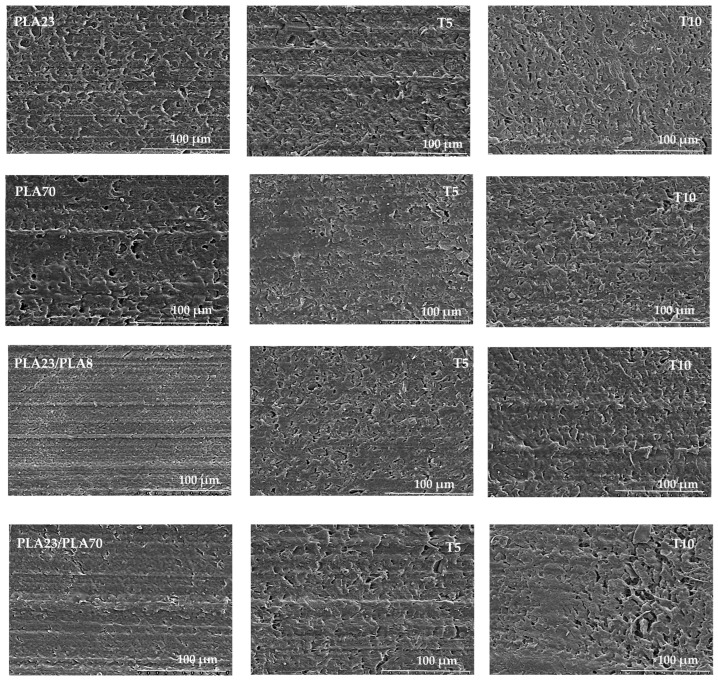
SEM micrographs of transverse cross-sections of injection-moulded TPS/BioPBS/PLA formulations at 1000× magnification.

The SEM micrographs of the transverse cross-sectional morphology of injection-moulded TPS/bioPBS/PLA formulations are shown in Figure 2. The micrographs are arranged in a 4 × 3 matrix, where rows correspond to different PLA grades or PLA blends (PLA23, PLA23/PLA8, PLA70, and PLA23/PLA70), and columns represent increasing talc content (0, 5, and 10 wt%). This configuration enables a systematic evaluation of the influence of PLA flow characteristics and talc loading on the internal morphology of the composites.

The PLA23 formulations exhibited a relatively heterogeneous internal morphology, particularly in the talc-free sample (T0), where the presence of voids and poorly defined domains was evident across the cross-section [27,28]. Upon talc incorporation (T5 and T10), the structure became progressively rougher and more textured, showing more pronounced microstructural irregularities likely associated with the presence of talc particles [29]. However, a further increase in surface roughness and structural heterogeneity was observed at higher talc contents, which may be related to the increased filler content within the matrix [30].

The PLA70 group showed generally dense cross-sectional morphologies, with limited visible porosity, reflecting the good flowability of this PLA grade during injection moulding [29]. At T0 and T5, the matrix appeared compact and continuous, with limited void formation. Nevertheless, at the highest talc content (T10), a marked increase in microstructural roughness was observed, with more pronounced surface irregularities and textural features [30,31]. This indicates that excessive talc loading significantly affects the internal architecture, even in systems with favourable flow behaviour.

In the PLA23/PLA8 series, an overall improvement in internal cohesion was observed. The T0 sample displayed a more continuous and flow-aligned morphology, indicative of efficient packing during injection. At moderate talc loading (T5), the cross-sections remained compact and relatively uniform, while at T10, although textural roughness increased, no significant voids or interfacial debonding were detected. This behaviour suggests that blending PLA grades with different flow characteristics may contribute to maintaining structural continuity even at higher talc loadings [32,33].

The PLA23/PLA70 formulations exhibited a relatively balanced internal morphology across the investigated talc contents. The T0 and T5 samples showed continuous and well-packed structures with evident flow-induced orientation [29]. At T10, although increased roughness and textural irregularities were present, the overall structural cohesion was preserved, with no clear signs of critical discontinuities or filler pull-out. This suggests that the combination of PLA grades with different MFI values may favour a relatively homogeneous filler distribution and maintain matrix continuity under the studied conditions [32,33].

Overall, the SEM cross-sectional analysis demonstrates that both PLA flow characteristics and talc loading play a role in defining the internal morphology of injection-moulded TPS/bioPBS-based blend and composites [27,30,33]. While increasing talc content tends to introduce textural roughness and microstructural heterogeneities, the use of PLA blends appears to help maintain matrix continuity and structural compactness, mitigating some of the morphological irregularities observed at higher filler loadings.

### 3.2. Chemical Structure

**Figure 3:**
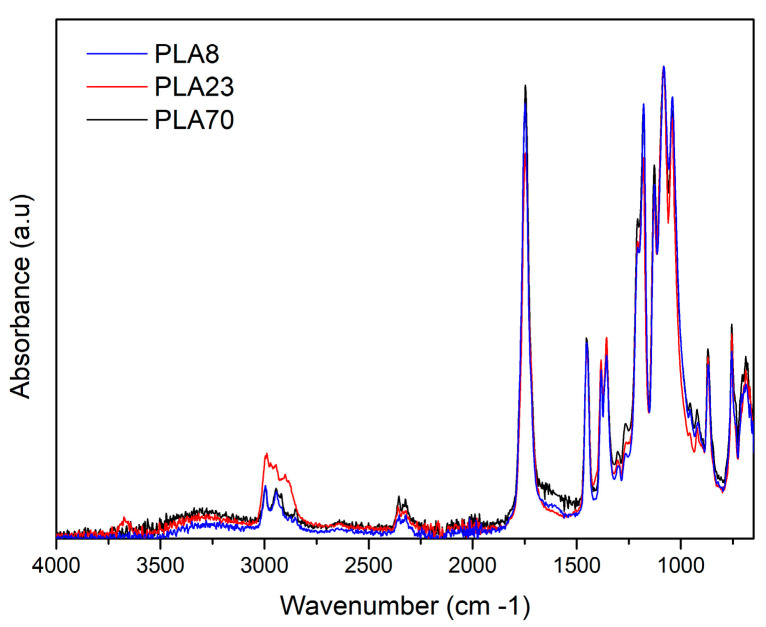
FTIR spectra of the PLA grades used in this study.

Before analysing the multicomponent TPS/BioPBS/PLA formulations, the FTIR spectra of the three PLA grades employed in this study (PLA8, PLA23 and PLA70) were examined in order to identify potential differences associated with their molecular characteristics. As shown in Figure 3, all PLA grades exhibited the characteristic absorption bands of polylactic acid, including the strong ester carbonyl (C=O) stretching band located at approximately 1710 cm^−1^, the C–O–C stretching vibrations in the 1180–1080 cm^−1^ region, and the C–H stretching bands of methyl groups around 2995–2945 cm^−1^ [28,34,35]. No additional absorption bands were detected, confirming that all grades share the same chemical structure. However, differences in band intensity and peak definition were observed among the spectra, particularly in the carbonyl, C–O–C, and C–H stretching regions (2850–3000 cm^−1^). These variations may be related to differences in the physical characteristics of the PLA grades, including their melt flow behaviour and molecular architecture, which are known to influence the processing and performance of PLA materials [32,33].

**Figure 4:**
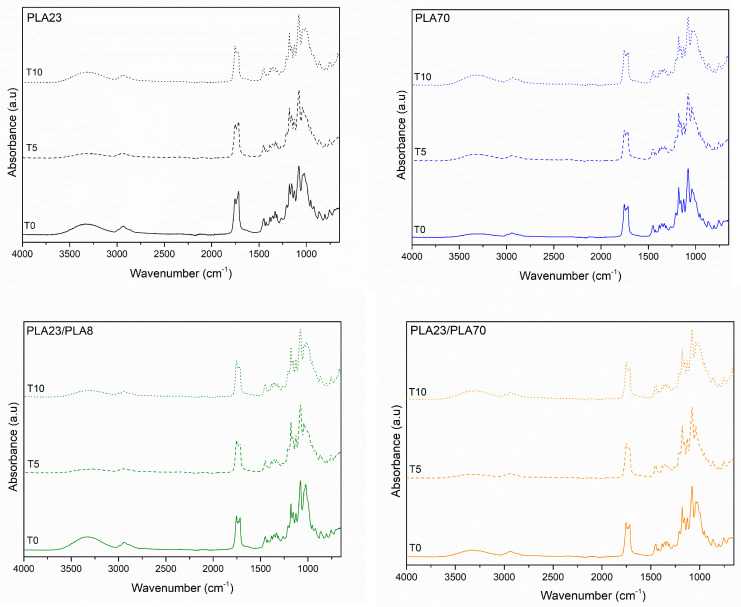
FTIR spectra of the TPS/BioPBS/PLA formulations grouped by PLA grade.

The FTIR spectra of the TPS/BioPBS/PLA formulations containing different PLA grades and PLA combinations, grouped according to their formulation strategy, are presented in Figure 4. All samples exhibited the characteristic absorption bands associated with the main components of the system [27,34]. A broad absorption band centred around 3400 cm^−1^ was observed in all formulations and attributed to O–H stretching vibrations from starch and glycerol [27,36]. The bands located at approximately 2920 and 2850 cm^−1^ correspond to C–H stretching vibrations of aliphatic methylene groups, mainly associated with BioPBS and PLA chains. A strong absorption band in the 1715–1750 cm^−1^ region was assigned to the ester carbonyl (C=O) stretching vibrations characteristic of PLA and BioPBS [34,37]. Additionally, the region between 1000 and 1200 cm^−1^ presented multiple absorption bands related to C–O and C–O–C stretching vibrations, mainly associated with starch and PLA [27,28].

For formulations containing a single PLA grade, noticeable differences in the intensity and definition of the ester carbonyl band were observed depending on the used PLA type [32,33]. Systems incorporating PLA23 exhibited a relatively broader carbonyl band compared to the other formulations, while maintaining the characteristic band position of PLA. In contrast, formulations containing PLA8 showed a more intense and better-defined C=O absorption band. However, no shift in band position was observed, indicating that these differences are related to variations in spectral intensity rather than to chemical modifications of the system. PLA70-containing systems presented intermediate spectral behaviour between PLA23 and PLA8 formulations, while preserving the characteristic absorption bands of PLA. In all cases, no additional absorption bands were detected, indicating the absence of chemical reactions between the PLA grades and the TPS/BioPBS matrix [27].

In formulations containing blends of different PLA grades, such as PLA23/PLA8 and PLA23/PLA70 combinations, a more balanced spectral profile was observed. These systems exhibited an increased definition of the carbonyl band compared to single-PLA formulations, while maintaining similar band positions. The absence of new absorption peaks confirms that the coexistence of PLA grades with different flow characteristics does not induce detectable chemical bonding between the components. The observed spectral differences are therefore attributed to physical blending effects rather than chemical modifications [27]. The enhanced definition observed in the 1180–1190 cm^−1^ region, attributed to C–O–C stretching vibrations of PLA, suggests a more homogeneous contribution of the PLA phase in these mixed systems.

The effect of talc content was consistent across all formulations. A progressive decrease in the intensity of the O–H stretching band was observed as the talc content increased from T0 to T10, indicating a reduction in free hydroxyl groups within the TPS phase [36,38]. This behaviour suggests an interaction between talc particles and the polymer matrix, reflected by the reduction in hydroxyl band intensity. No evidence of new chemical bonds associated with talc incorporation was detected [38,39]. No new bands associated with chemical interactions involving talc were detected, confirming its role as a physically interacting filler rather than a reactive agent [38].

Overall, the FTIR results demonstrate that the incorporation of different PLA grades and their combinations modify the vibrational response of the TPS/BioPBS matrix mainly through changes in band intensity and definition, without altering the chemical structure of the system [27,34].

### 3.3. Thermal Properties

**Figure 5:**
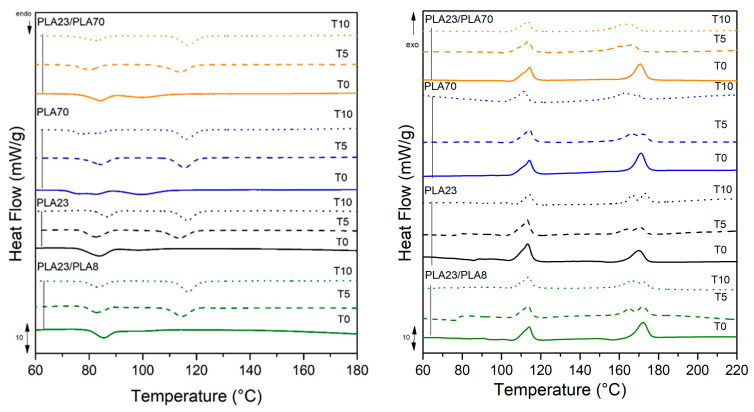
DSC of cooling (**left**) and second heating curves (**right**) of TPS/PBS/PLA/talc blends and composites.

Differential Scanning Calorimetry (DSC) was used to investigate the thermal transitions and crystallisation behaviour of the TPS/PBS/PLA blends, as well as the influence of PLA grade and talc content [40]. The DSC thermograms are presented in Figure 5, while the characteristic thermal parameters are summarised in Table 2.

During the cooling step, all formulations exhibited a well-defined crystallisation peak associated with the PBS phase, with crystallisation temperatures (T_c_,PBS) ranging between approximately 80 and 86 °C [41]. No clear systematic trend in T_c_,PBS was observed with increasing talc content across the different PLA systems. For instance, in TPS/PBS–PLA23 formulations, T_c_,PBS increased slightly from 83.32 ± 0.66 °C (T0) to 83.63 ± 4.07 °C (T10), whereas a gradual decrease was observed in the PLA23/PLA8 and PLA23/PLA70 systems. These variations remain relatively small and suggest that the incorporation of talc has only a limited influence on the crystallisation temperature of the PBS phase under the studied conditions.

The crystallisation enthalpy of PBS (Δh_c_,PBS) showed a moderate decrease with increasing talc content, suggesting the formation of finer and more numerous crystalline structures rather than an increase in overall crystallinity [8]. This reduction in Δh_c_,PBS may be attributed to restricted chain mobility induced by the presence of rigid mineral particles and the partial confinement of PBS chains within the multiphase TPS matrix [8,37].

In contrast, PLA crystallisation during cooling was strongly dependent on both the PLA grade and talc content. PLA-containing formulations exhibited crystallisation peaks at higher temperatures (T_c_,PLA), typically between 99 and 116 °C [38], with a marked increase in Δh_c_,PLA upon talc addition [42,43]. For example, in TPS/PBS–PLA70 formulations, Δh_c_,PLA increased from 9.38 ± 0.59 J/g for T0 to 15.47 ± 0.15 J/g for T5, confirming the strong nucleating effect of talc on PLA crystallisation. Similar nucleating effects of talc in PLA/PBS systems processed by melt technologies were previously reported by Pivsa-Art et al. [42] who also observed accelerated crystallisation together with increased stiffness and reduced ductility. Similar trends were observed in blended PLA systems (PLA23/PLA8 and PLA23/PLA70), where talc promoted earlier and more intense PLA crystallisation, particularly for higher-melt-flow PLA grades [38].

During the second heating cycle, all samples exhibited a melting endotherm corresponding to PBS, with melting temperatures (T_m_,PBS) centred around 112–114 °C and only minor variations as a function of formulation [41]. The relative stability of T_m_,PBS also suggested that neither PLA grade nor talc content significantly altered the crystalline structure of PBS formed during cooling.

A second melting peak associated with the PLA phase was observed in all PLA-containing formulations, with T_m_,PLA values ranging between approximately 164 and 172 °C [38]. The presence of talc slightly modified the melting behaviour of PLA, generally leading to a modest decrease in T_m_,PLA at higher talc contents, particularly for PLA70-based systems [43]. This effect may be related to the formation of thinner or less perfect PLA crystals due to accelerated nucleation and faster crystallisation kinetics induced by talc [43].

The melting enthalpy of PBS (Δh_m_,PBS) systematically decreased with increasing talc and PLA content, from −18.22 ± 2.73 J/g in TPS/PBS–PLA23–T0 to −9.05 ± 2.73 J/g in TPS/PBS–PLA23/PLA8–T10, indicating a reduction in PBS crystalline fraction within the multiphase blends [37]. Conversely, PLA melting enthalpies (Δh_m_,PLA) remained relatively stable across formulations, suggesting that talc primarily affects the crystallisation process rather than the final melting behaviour of PLA crystals [38,43].

Overall, the DSC results indicate that talc acted as an efficient nucleating agent for the PLA phase with a more pronounced effect on PLA crystallisation, particularly for higher-flow PLA (lower molar mass) grades [42,43].

The combined influence of PLA grade and talc content governs the balance between crystallisation kinetics and crystalline perfection, in good agreement with the morphological trends observed by SEM and the mechanical behaviour discussed in the following section [37]. These thermal modifications play a key role in tailoring the stiffness and thermal stability of TPS/PBS/PLA injection-moulded systems [40].

### 3.4. Mechanical Performance

#### 3.4.1. Mechanical Behaviour Under Tensile Loading

**Figure 6:**
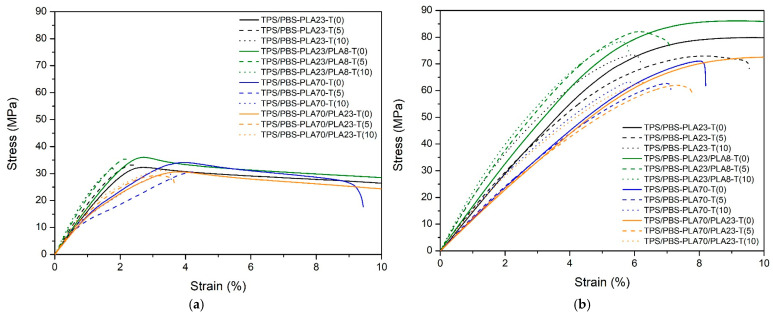
Mechanical behaviour of TPS/PBS/PLA formulations with varying PLA grades and talc contents: (**a**) tensile stress–strain curves; (**b**) flexural force–deflection curves.

The mechanical performance of the injection-moulded TPS/PBS/PLA blends was strongly affected by the PLA grade, PLA content and talc-free formulation, as shown in Figure 6a (stress–strain curves) and Table 3 [12,42]. Unlike the highly ductile behaviour observed in film samples processed by extrusion, all injection-moulded materials exhibited a predominantly elastic–plastic response with limited elongation at break, which is characteristic of semi-crystalline polymer blends processed under high cooling rates [41].

The reference TPS/PBS-PLA23 formulation exhibited moderate stiffness and strength, with a relatively low elongation at break (below 10%), reflecting the restricted chain mobility imposed by the injection moulding process [28,41]. The stress–strain curves show a well-defined yield point followed by a short plastic deformation region and subsequent strain softening, indicating limited energy dissipation prior to fracture [38].

When PLA8 was incorporated into the TPS/PBS-PLA23 system, a clear increase in stiffness was observed. The Young’s modulus increased noticeably, and the maximum stress values exceeded those of the PLA23 reference, indicating improved load-bearing capacity [42]. This behaviour can be attributed to the higher rigidity of the PLA8 phase and its contribution to stress transfer within the TPS/PBS matrix [32]. This is in agreement with the results reported by Dmitruk et al. [44], who observed that the incorporation of PBS and TPS into PLA-based injection-moulded systems significantly modified stiffness, tensile strength and deformation behaviour depending on blend composition and processing conditions. Their results also demonstrated that improvements in ductility are generally accompanied by reductions in modulus and strength, in agreement with the mechanical balance observed in the present study. However, this reinforcement was accompanied by a slight reduction in strain at break, suggesting a more constrained deformation mechanism [45].

In contrast, blends containing PLA70 displayed a different mechanical response. Although PLA70 formulations showed comparable or slightly lower yield stresses than PLA8-containing systems, their stress–strain curves revealed a more progressive plastic deformation and delayed strain softening. This behaviour is consistent with the higher melt flow index of PLA70, which likely promotes improved dispersion and interfacial adhesion within the blend during processing [1]. As a result, TPS/PBS-PLA70 formulations achieved a balanced combination of strength and limited ductility under tensile loading [12]. Comparable behaviour has been reported for PLA/PBSA systems, where the incorporation of more ductile aliphatic polyesters promoted improved elongation and stress redistribution while partially reducing stiffness and tensile strength [46].

Interestingly, hybrid systems combining PLA23 and PLA70 exhibited intermediate behaviour. These formulations showed reduced stiffness compared to PLA8-based blends but improved strain tolerance relative to single-PLA systems [12,32]. This suggests that blending PLA grades with different molecular characteristics can partially mitigate the brittleness typically associated with injection-moulded TPS-based materials [28].

#### 3.4.2. Mechanical Behaviour Under Flexural Loading

Flexural tests provided complementary information on the stiffness-dominated mechanical response of the TPS/PBS/PLA blends. The force–deflection curves presented in Figure 6b demonstrate a pronounced elastic regime followed by a gradual reduction in load-bearing capacity, without catastrophic failure within the tested deflection range [38].

The TPS/PBS-PLA23 reference formulation exhibited moderate flexural strength and stiffness, consistent with its tensile behaviour. The incorporation of PLA8 led to a significant increase in maximum flexural force and flexural modulus, confirming the reinforcing role of this PLA grade under bending loads [32]. The higher resistance to deformation under flexural stress highlights the effectiveness of PLA8 in enhancing rigidity in applications dominated by bending rather than tensile deformation [42].

Conversely, PLA70-containing formulations displayed slightly lower maximum flexural forces but more stable post-peak behaviour, indicating improved resistance to crack propagation and localised failure [1]. This trend is consistent with the tensile results and supports the hypothesis that PLA70 enhances stress redistribution within the matrix due to better processability and phase continuity [12].

Hybrid PLA23/PLA70 systems again showed intermediate performance, achieving a compromise between flexural strength and deformation tolerance [12,32]. These results suggest that tailoring the PLA grade composition offers a viable strategy to tune mechanical performance for injection-moulded applications requiring a balance between stiffness and damage tolerance [28].

#### 3.4.3. Heat Deflection Temperature (HDT)

Regarding the thermomechanical performance, the Heat Deflection Temperature (HDT) values (Table 3) remained relatively constant for all formulations, ranging between 47 and 49 °C. Neither the PLA grade nor the talc content produced a significant improvement in HDT, despite the noticeable increase in stiffness observed in both tensile and flexural properties. Similar behaviour has been reported by Aliotta et al. [47], who demonstrated that increases in stiffness induced by talc nucleation do not necessarily translate into substantial HDT improvements unless high crystallinity levels are achieved during injection moulding. This indicates that the reinforcement provided by PLA and talc is not sufficient to effectively restrict polymer chain mobility under thermal and mechanical loading conditions.

The limited variation in HDT suggests that the thermomechanical behaviour is primarily governed by the TPS/PBS matrix, whose relatively low thermal resistance dominates the response of the blends. Even in formulations with higher stiffness, the material softens at similar temperatures, highlighting a decoupling between room-temperature mechanical performance and high-temperature dimensional stability.

These results are consistent with the typical behaviour of TPS-based systems, where improvements in stiffness do not necessarily translate into enhanced heat resistance, representing a key limitation for applications requiring thermal stability under load.

## 4. Conclusions

This work systematically investigated the influence of PLA grade and talc content on the structure–property relationships of injection-moulded TPS/BioPBS/PLA/talc blends and composites, for rigid and semi-rigid applications. The combinations of pairs of PLA grades with different MFI were considered, together with the additives of talc at 5 and 10%.

Morphological analysis revealed that PLA grade strongly affected phase continuity and internal cohesion. High-flow PLA grades promoted improved dispersion and more homogeneous fracture surfaces, whereas excessive talc contents introduced localised microstructural heterogeneities unless compensated by adequate PLA selection. Hybrid PLA formulations with different PLA grades and flowability provided a balanced morphology.

FTIR analysis revealed slight variations in band intensity and definition among formulations, while characteristic absorption bands remained unchanged. No evidence of new chemical bonds or modifications was thus detected, indicating that the observed differences among formulations were mainly related to physical blending effects rather than chemical interactions.

Thermal analysis demonstrated that talc acted as an effective heterogeneous nucleating agent, particularly for the PLA phase, slightly increasing crystallisation temperature while reducing crystallisation enthalpy due to constrained chain mobility. PLA crystallisation was strongly influenced by both grade and filler content, confirming that nucleation kinetics and crystal perfection can be tuned through formulation design. Moreover, melting temperatures remained relatively stable, indicating that talc primarily modified crystallisation behaviour rather than crystal stability.

Mechanical performance was predominantly governed by PLA grade rather than PLA content alone. PLA8 acted as a stiffening agent, increasing Young’s modulus, tensile strength and flexural modulus, while reducing ductility. In contrast, PLA70 promoted more progressive deformation and improved stress redistribution, leading to enhanced damage tolerance despite lower stiffness. Hybrid PLA systems enabled partial mitigation of brittleness, demonstrating that combining grades is an effective strategy to balance rigidity and deformation capacity.

Overall, the results confirm that the simultaneous optimisation of PLA grade and talc content provides a versatile design approach for tailoring TPS/BioPBS-based injection-moulded materials. The study establishes practical formulation guidelines for achieving controlled stiffness, thermal stability and mechanical performance in biodegradable systems intended for rigid and semi-structural applications, without relying on reactive compatibilisers or complex chemical modifications. These materials show potential for rigid and semi-rigid biodegradable applications manufactured by injection moulding, such as trays, lids, disposable products and low-demand technical components. Furthermore, the possibility of tailoring stiffness and deformation behaviour through PLA grade selection and talc incorporation provides useful design criteria for application-specific compostable packaging.

## Figures and Tables

**Table 1:** Composition (%wt) and labelling of TPS/BioPBS/PLA blends. Note: PLA labelled in relationship with its flowability (MFI), as detailed in Section 2.1.

Reference	TPS	BioPBS	PLA23	PLA8	PLA70	Talc
TPS/PBS-PLA23-T(0)	36.5	25	38.5	0	0	0
TPS/PBS-PLA23-T(5)	36.5	22	36.5	0	0	5
TPS/PBS-PLA23-T(10)	36.5	19	34.5	0	0	10
TPS/PBS-PLA23/PLA8-T(0)	36.5	25	23.5	15	0	0
TPS/PBS-PLA23/PLA8-T(5)	36.5	22	21.5	15	0	5
TPS/PBS-PLA23/PLA8-T(10)	36.5	19	19.5	15	0	10
TPS/PBS-PLA23/PLA70-T(0)	36.5	25	15	0	23.5	0
TPS/PBS-PLA23/PLA70-T(5)	36.5	22	15	0	21.5	5
TPS/PBS-PLA23/PLA70-T(10)	36.5	19	15	0	19.5	10
TPS/PBS-PLA70-T(0)	36.5	25	0	0	38.5	0
TPS/PBS-PLA70-T(5)	36.5	22	0	0	36.5	5
TPS/PBS-PLA70-T(10)	36.5	19	0	0	34.5	10

**Table 2:** DSC results (crystallisation temperatures *Tc*, crystallisation enthalpies Δ*hc*, melting temperatures *Tm* of PBS and PLA, and melting enthalpies Δ*hm* of PBS and PLA) for TPS/PBS/PLA/talc blends and composites.

	Cooling				Second Heating			
Blend	T_C_,PBS (°C)	Δh_C_,PBS (J/g)	T_C_,PLA (°C)	Δh_C_,PLA (J/g)	T_m_,PBS (°C)	T_m_,PLA (°C)	Δh_m_,PBS (J/g)	Δh_m_,PLA (J/g)
TPS/PBS-PLA23-T(0)	83.32 ± 0.66	−12.565 ± 0.86	99.01 ± 0.01	2.37 ± 0.15	112.09 ± 1.75	168.72 ± 1.70	18.22 ± 2.73	−15.03 ± 0.58
TPS/PBS-PLA23-T(5)	83.59 ± 1.67	−10.84 ± 1.09	113.49 ± 0.19	12.41 ± 0.77	113.28 ± 0.17	171.18 ± 0.68	13.78 ± 1.53	−11.34 ± 0.19
TPS/PBS-PLA23-T(10)	83.63 ± 4.07	−8.36 ± 1.28	115.65 ± 1.42	13.89 ± 1.45	112.96 ± 1.88	167.31 ± 8.28	9.85 ± 2.46	−12.55 ± 0.34
TPS/PBS-PLA23/PLA8-T(0)	85.88 ± 0.72	−10.28 ± 0.29	101.21 ± 1.07	1.74 ± 1.52	114.03 ± 0.08	172.13 ± 0.01	11.92 ± 0.33	−17.39 ± 0.09
TPS/PBS-PLA23/PLA8-T(5)	84.75 ± 2.17	−8.97 ± 1.04	115.25 ± 0.43	14.38 ± 0.87	113.99 ± 0.48	172.68 ± 1.32	11.11 ± 0.32	−14.14 ± 1.16
TPS/PBS-PLA23/PLA8-T(10)	82.15 ± 1.52	−7.59 ± 2.20	116.20 ± 0.94	12.37 ± 3.68	114.18 ± 0.44	166.49 ± 0.13	9.05 ± 2.73	−13.51 ± 3.36
TPS/PBS-PLA23/PLA70-T(0)	83.82 ± 0.31	−8.94 ± 0.06	99.61 ± 0.25	6.36 ± 0.89	114.31 ± 0.07	170.97 ± 0.29	13.02 ± 0.14	−17.61 ± 2.07
TPS/PBS-PLA23/PLA70-T(5)	80.19 ± 0.16	−9.64 ± 0.37	113.89 ± 0.13	14.31 ± 0.94	113.49 ± 0.22	165.17 ± 1.03	12.49 ± 0.35	−17.19 ± 0.15
TPS/PBS-PLA23/PLA70-T(10)	80.72 ± 2.56	−9 ± 0.27	116.35 ± 0.81	14.82 ± 0.28	113.42 ± 0.01	163.57 ± 0.84	10.93 ± 0.42	−16.29 ± 0.14
TPS/PBS-PLA70-T(0)	82.55 ± 0.17	−9.24 ± 0.42	99.79 ± 0.05	9.38 ± 0.59	114.17 ± 0.11	170.73 ± 0.47	14.36 ± 0.05	−17.36 ± 0.26
TPS/PBS-PLA70-T(5)	84.07 ± 0.38	−10.70 ± 0.03	115.25 ± 0.39	15.47 ± 0.15	114.16 ± 0.06	167.38 ± 2.65	12.69 ± 0.24	−15.61 ± 0.67
TPS/PBS-PLA70-T(10)	80.29 ± 4.62	−10.37 ± 0.17	116.76 ± 0.43	13.93 ± 0.48	112.30 ± 1.16	163.95 ± 1.14	10.72 ± 0.21	−13.21 ± 1.69

**Table 3:** Mechanical properties (tensile strength, Young’s modulus, elongation at break, flexural stress, strain and modulus) of TPS/PBS/PLA formulations with different PLA grades and talc contents.

Blend	Young’s Modulus (GPa)	Tensile Strength at Break (MPa)	Elongation at Break (%)	Flexural Modulus (GPa)	Flexural Strain (%)	Flexural Stress (MPa)	Heat Deflection Temperature, HDT (°C)
TPS/PBS-PLA23-T(0)	1.8 ± 0.1	34 ± 1	16 ± 3	1.5 ± 0.2	5.6 ± 0.1	48 ± 0	49
TPS/PBS-PLA23-T(5)	2.2 ± 0	32 ± 1	2 ± 0	1.6 ± 0.2	4.6 ± 0.1	43 ± 0	48
TPS/PBS-PLA23-T(10)	2.6 ± 0.1	31 ± 1	2 ± 0	1.6 ± 0.2	3.4 ± 0.1	44 ± 1	48
TPS/PBS-PLA23/PLA8-T(0)	1.8 ± 0.1	36 ± 1	35 ± 7	1.7 ± 0.2	5.3 ± 0.1	51 ± 1	49
TPS/PBS-PLA23/PLA8-T(5)	2.4 ± 0.1	34 ± 1	2 ± 0	2.1 ± 0.2	3.6 ± 0.1	49 ± 1	48
TPS/PBS-PLA23/PLA8-T(10)	2.7 ± 0.1	32 ± 1	2 ± 0	2.4 ± 0.3	3.1 ± 0.1	47 ± 1	49
TPS/PBS-PLA23/PLA70-T(0)	1.4 ± 0.1	33 ± 1	22 ± 7	1.1 ± 0.2	5.5 ± 0.8	42 ± 4	48
TPS/PBS-PLA23/PLA70-T(5)	1.6 ± 0	29 ± 1	3 ± 0	1.3 ± 0	4.1 ± 0.1	37 ± 0	47
TPS/PBS-PLA23/PLA70-T(10)	1.9 ± 0	31 ± 1	3 ± 0	1.7 ± 0	3.6 ± 0.1	39 ± 1	47
TPS/PBS-PLA70-T(0)	1.4 ± 0	34 ± 1	8 ± 1	1.2 ± 0	4.6 ± 0.1	42 ± 0	48
TPS/PBS-PLA70-T(5)	1.5 ± 0.1	31 ± 1	4 ± 0	1.3 ± 0	3.8 ± 0.2	36 ± 1	47
TPS/PBS-PLA70-T(10)	1.6 ± 0.2	29 ± 2	3 ± 0	1.6 ± 0.1	3.3 ± 0.1	37 ± 0	47

## Data Availability

The original contributions presented in this study are included in the article. Further inquiries can be directed to the corresponding authors.

## Abbreviations

TPS, thermoplastic starch; PBS, poly(butylene succinate); PLA, poly(lactic acid); PBAT, poly(butylene adipate-co-terephthalate); PBSA, poly(butylene succinate-co-adipate); MFI, melt flow index; SEM, scanning electron microscopy; FTIR, Fourier-transform infrared spectroscopy; ATR, attenuated total reflectance; DSC, differential scanning calorimetry; HDT, heat deflection temperature.
